# Supplementary material for: Severe obesity as an oligogenic condition: evidence from 1714 adults seeking treatment in the UK National Health Service
Source: Hum Genomics. 2025 Dec 19;20:20. doi: 10.1186/s40246-025-00895-7 (PMC12849414; doi:10.1186/s40246-025-00895-7)
Supplement: Supplementary file 2 — Supplementary Material 2. [file 40246_2025_895_MOESM2_ESM.pdf]

Imperial College London

Prof Alexandra I F Blakemore
Commonwealth Building 5.S5a
Hammersmith Hospital
Hammersmith Campus
Du Cane Road, London W12 0NN

Participant Consent Form for NHS HOSPITAL TRUSTS - POST SURGERY

Title of project: Personalised Medicine for Morbid Obesity

Name of Principal Investigator: Prof A. Blakemore. Please initial each statement:

1. I confirm that I have read and understand the Participant Information Sheet Protocol Version 12 dated 20th Oct 2016 for the above study. ☐

2. I have had the opportunity to ask questions and discuss this study. All my questions have been answered fully and I have received enough information about the study. ☐

3. I understand that my participation is voluntary and that I am free to withdraw at any time, without giving any reason, and without my medical care or legal rights being affected. ☐

4. I agree that my medical notes and data collected from the study may be accessed by responsible individuals involved in the study; Imperial College London, Imperial College Healthcare NHS Trust or other Trusts, , or by regulatory authorities where it is relevant to my taking part in this research. ☐

5. I give permission for my data to be used for research by individuals involved in the study and Imperial College Healthcare NHS Trust so long as they do not contain identifying personal information. ☐

6. I give permission for the blood test results collected upon my initial referral to the service to be used for the purposes of the study. ☐

7. I give permission for my GP to be informed of my participation in this study and the results of any medical tests from my visits. ☐

8. I agree for a DNA sample to be taken and stored to look for changes that may be involved in obesity and the control of appetite. This may include sending my anonymised sample to other research centres in or outside the UK and may include commercial companies. ☐

9. I give permission for anonymised data on my exercise and mood, recorded through the smartphone apps Moves and WLCompanion, to be used in this study ☐

10. I give permission for the data collected in the questionnaires and smell test to be used for the purposes of the study. ☐

11. I agree to my samples being collected as detailed in the patient information sheet. ☐

12. The indemnity arrangements have been discussed with me. ☐

13. I agree to take part in the above study. ☐

14. I am happy to be contacted for possible participation in future research studies. ☐

______________________	______________	______________
Name of Participant (block capitals)	Signature	Date
______________________	______________	______________
Principal Investigator	Signature	Date
______________________	______________	______________
Name of Person taking consent<br>(if different from Principal Investigator)	Signature	Date
